# To What Extent Local Forest Soil Pollen Can Assist Restoration in Subtropical China?

**DOI:** 10.1038/srep37188

**Published:** 2016-11-18

**Authors:** Zhongyu Sun, Jun Wang, Hai Ren, Qinfeng Guo, Junwu Shu, Nan Liu

**Affiliations:** 1 Guangdong Open Laboratory of Geospatial Information Technology and Application, Guangzhou Institute of Geography, Guangzhou, 510070, China; 2 Key Laboratory of Vegetation Restoration and Management of Degraded Ecosystems, South China Botanical Garden, Chinese Academy of Sciences, Guangzhou, 510650, China; 3 Eastern Forest Environmental Threat Assessment Center, USDA Forest Service, RTP, NC 27709, USA; 4 State Key Laboratory of Paleobiology and Stratigraphy, Nanjing Institute of Geology and Palaeontology, Chinese Academy of Sciences, Nanjing, 210008, China

## Abstract

Long-term ecological data play a vital role in ecological conservation and restoration, however, using information from local forest soil pollen data to assist restoration remains a challenge. This study analyzed two data sets, including 1) surface soil pollen (0–5 cm) and current vegetation data from four near-natural communities and four plantations, and 2) fossil pollen from soil profiles (0–80 cm) from a regional climax community and a degraded land. The pollen representativeness and similarity indexes were calculated. The results showed a low similarity between soil pollen and current vegetation (about 20%) thus forest soil pollen data should be used with caution when defining reference ecosystems. Pollen from *Gironniera* and Rutaceae which were abundant in broadleaved forest, were also detected in the 40–80 cm layer of a soil profile from the degraded land, which indicates its restoration possibility. Our study considered that the early restoration stage of the study area may benefit from using plant taxa of *Pinus*, Poaceae, *Lonicera, Casuarina, Trema* and *Quercus*. As *Pinus, Castanopsis, Gironniera*, Rutaceae, *Helicia, Randia*, Poaceae, *Dicranopteris* and *Pteris* always existed during succession, for regional forest restoration under global climate change, the roles of such “stable species” should be considered.

Information based on long-term ecological data plays a vital role in ecological conservation and restoration^1^,^2^,^3^. Most ecologists now realize that establishing realistic ecosystem baseline or reference ecosystem requires understanding of the historical range of natural variability of the vegetation as well as climate and disturbance regimes^4^. A reference ecosystem serves as a model for planning restoration and for the subsequent evaluation of restoration success. However, selecting an appropriate reference ecosystem is still a major challenge mostly due to a lack of critical information^5^. A series of characteristics of a historically undisturbed ecosystem could be used to define a reference ecosystem^6^. Unfortunately, very few historical or undisturbed ecosystems still exist because of human disturbance and global change^7^. Some of the information required to reconstruct a historical or original undisturbed ecosystem, however, can be partly obtained from fossil pollen^8^. Pollen data, nevertheless, must be used with caution because pollen stored in soils can be altered by storage environment conditions^9^, such as soil characteristics and microenvironments^10^. Pollen representativeness in any deposit may be also conditioned by differential production and transport of the different pollen types^11^, as well as ground litter and eluviation in forests^12^. Thus, extracting information for reference ecosystems from soil pollen remains a major task. The modern pollen deposition and pollen-vegetation relation are frequently used to improve interpretation of fossil pollen samples^13^,^14^.

Latosols are widely distributed in South China, where the soil pH is low and eluviation is common. Most fossil pollen samples previously collected in this region were from moss, swamps, wetlands, and lakes^15^,^16^,^17^, because the pollen storage environments in previous medium were relatively stable and less disturbed. However, pollen in sediments of swamps, wetland or lakes contain complex community information of the whole region. Therefore, although the information benefits to reconstructing the paleoclimate of the research region, for the local restoration, the information are generally not sufficient and precise enough. Without accurate local fossil pollen data, a climax community, monsoon evergreen broadleaved forest, was previously used as the reference ecosystem for local restoration in subtropical China^18^. The reference ecosystem also neglects the possible effects of local microenvironment and history^19^. This leads to a serious question, that is, whether the pollen stored in the soil under communities can provide complementary assistance for local restoration.

To date, most studies on fossil pollen or modern pollen have been performed on regional scales. Here, we evaluate the applicability of local forest soil pollen data for selecting reference ecosystems in South China. Specifically, we examined the relationships between the surface soil pollen and current vegetation in four near-natural forest communities and in four plantations in subtropical China. We select one 400 years old near-natural growing community and a degraded bare land to examine how pollen in the soil profile can help researchers identify the reference ecosystem for restoration. We attempted to address the following issues in subtropical China: (1) What is the relationship between the surface soil pollen and the current vegetation? And (2) to what extent the forest soil pollen can assist in selecting reference ecosystem?

## Results

### The composition of surface soil pollen

A total of 41 pollen taxa were detected in the surface soils in the near-natural communities (24 trees, eight herbs, and nine ferns), and 32 pollen taxa were identified in the surface soils in the plantations (19 trees, five herbs, and eight ferns). Palynological richness was higher in near-natural communities than in the plantations, but pollen from tree species represented 59% of all pollen in both near-natural communities and plantations.

**Figure 1:**
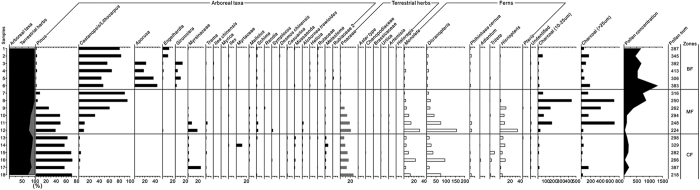
Surface soil pollen diagram in natural communities CF, MF, and BF at the Dinghushan Natural Reserve in South China. Note: BF = broadleaved forest; MF = pine and broadleaved mixed forest; CF = coniferous forest. The Y axis on the left indicates the sample number.

In coniferous forest, the dominant surface soil pollen types were from *Pinus*, Poaceae, and *Dicranopteris* (Table 1 and Fig. 1). In pine and broadleaved mixed forest and broadleaved forest, the dominant pollen types were *Castanopsis*, Poaceae, and *Dicranopteris*.

**Figure 2:**
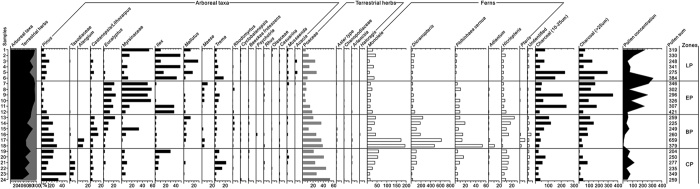
Surface soil pollen diagram in plantations in South China. Note: LP = legume plantation; EP = eucalyptus plantation; BP = broadleaved plantation; CP = coniferous plantation. The Y axis on the left indicates the sample number.

**Figure 3:**
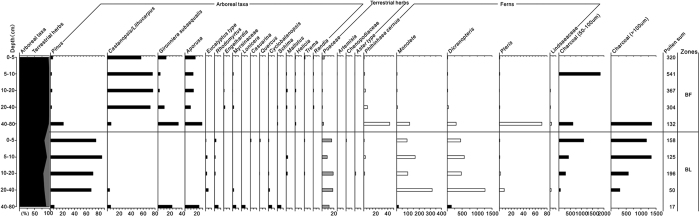
Pollen profile at broadleaved forest and bare land in Guangdong, South China. Note: BF = broadleaved forest (regional climax); BL = bare land. The Y axis on the left indicates soil depth (cm).

The dominant pollen in the surface soil at coniferous plantation and broadleaved plantation was from *Pinus*, Poaceae, and *Dicranopteris* (Table 1 and Fig. 2). In contrast to coniferous plantation, pollen from Myrsinaceae and *Castanopsis* reached 8.9% and 8.2% in broadleaved plantation, respectively (unless noted otherwise, the percentages were relative to the total tree and herb pollen in the sample). At the same time, pollen taxa from *Trema* and Poaceae in broadleaved plantation were 2.5% and 29.6%, respectively (Fig. 2). The pollen spectrum of Eucalyptus plantation was characterized by abundant pollen from *Eucalyptus* (17.2%) and Myrsinaceae (39.3%) and a low content of *Pinus* (3.9%). In addition, pollen taxa from *Ilex* (12.6%), *Trema* (4.5%), and *Melastoma* (2.5%) were also observed in Eucalyptus plantation (Fig. 2). The pollen assembly of legume plantation was characterized by the occurrence of pollen from *Acacia* and abundant pollen from *Dicranopteris* (40.2%). Pollen taxa *Ilex* (29.2%), *Mallotus* (11.2%), Myrsinaceae (16.0%), and *Trema* (6.3%) were also detected in legume plantation (Fig. 3).

### Pollen profiles

The pollen profiles of regional climax vegetation and degraded bare lands are shown in Fig. 3. The ^14^C dating age of soils was listed in Table 2. The broadleaved forest was characterized by a high content of pollen from *Castanopsis* and *Lithocarpus* in the 0–40 cm layer of the soil profile (Fig. 3). Other common pollen included those from *Gironniera*. The content of pollen from Poaceae was low. In the 40–80 cm layer, the proportions of pollen from *Pinus*, Poaceae, and *Dicranopteris* were 21.2, 2.3, and 295.5%, respectively. These data indicated that broadleaved forest was a typical secondary forest. In the 0–40 cm layer, the dominant pollen was from *Castanopsis* and *Lithocarpus*, representing a succession stage from a *Pinus* forest to an evergreen broadleaved forest.

The dominant pollen taxa in all layers of the soil profile in bare land were from *Pinus* and Poacea. Pollen from *Dicranopteris* was also abundant. In the top four layers at bare land (from 0 to 40 cm depth), pollen from *Pinus* was still dominant. Pollen taxa Poaceae and *Dicranopteris* were common in these layers. A small amount of pollen of *Rhodomyrtus* were also detected. In the bottom layer (40–80 cm), pollen from *Pinus* was less than 10%, and pollen from *Gironniera* and Rutaceae (about 20%) was more abundant. This layer also contained pollen from *Rhodomyrtus*, Myrsinaceae, *Lonicera, Cyclobalanopsis*, and *Schima* (about 5% for each). This suggests that some broadleaved trees or shrubs existed in this area.

### The relationship between soil pollen data and current vegetation

The representativeness (as indicated by R values) of the major types of pollen are listed in Table 3. The major pollen in the surface soil (0–5 cm) was identified to 26 taxa, of which 19 were trees or shrubs, four were ferns, and three were herbs. The lowest R value was 0.02 from *Ficus*, and the highest was 33.67 from *Acacia*. The average R values from *Castanopsis*/*Lithocarpus, Trema, Acacia*, Poaceae and *Engelhardia* were >10. The R values of a given taxon varied among the communities.

The similarities between soil pollen and vegetation as indicated by the Sorensen and Jaccard indices are shown in Table 4. The average *SI* value was 0.201, which was slightly higher in near-natural communities (0.215) than in artificial communities (0.190). The average *C*_*J*_ value was 0.112, and the *C*_*J*_ value was also slightly higher in near-natural communities (0.121) than in artificial communities (0.105). The *C*_*J*_ and *SI* values did not significantly differ between near-natural forests and plantations (*t*-test, *P* = 0.05) but were higher in the coniferous forest and the legume plantation than in the other communities. The average CJ and SI values, however, were relatively low in this study area.

## Discussion

### The soil pollen composition

Our results show that pollen is abundant in the surface soils (0–5 cm depth) of both near-natural communities and plantations in South China. The number of pollen types in the surface soils is higher in near-natural communities than in plantations, probably due to the structural difference between the two types of communities. The aboveground plant taxon richness is higher and the structure is more complex in the near-natural plant communities than in the plantations^20^.

The surface soil pollen spectra also differ between near-natural communities and plantations. The pollen spectra of coniferous forest, pine and broadleaved mixed forest, and broadleaved forest were dominated by *Pinus*, Poaceae, or *Castanopsis*, and other pollen represented <0.05% of the pollen spectra. In plantations, however, the pollen of other taxa such as *Ilex, Maesa, Trema*, and *Acacia*, each represented about 10% of the total tree and herb pollen. This difference between near-natural communities and plantations may be caused by the differences in the depth of the litter layer, soil pH, soil chemical characteristics or other aspects of the environment^21^. The litter layer was thicker in the near-natural communities than in plantations, and this thicker layer would likely prevent some pollen from entering the soil. The generally higher humidity in near-natural forests than in plantations might also have accelerated the decay of some pollen^10^. Finally, gaps are more abundant in plantations than in near-natural forests and may have allowed more pollen entering the soil.

In the pollen profiles in soil layers from 0–80 cm, a large number of pine pollen was found at 40–80 cm in broadleaved forest. The decrease in the percentage of pine pollen and increase of broadleaved forest pollen with decreasing soil depth indicate the actual succession from pine forest to broadleaved forest^22^. It was known that the broadleaved forest was originally a pine forest planted by the Buddhist of Qingyun temple nearly 400 years ago. Thereafter, the forest started succession under a near-natural condition, and now develops into a broadleaved forest. In addition, pollen from broadleaved taxa such as *Gironniera* and Rutaceae were detected in the bottom soil profile of bare land. ^14^C dating age indicates that the pollen had been stored from 2375–1130 years BP (Table 2), indicating the existence of some broadleaved trees or shrubs in this period. After this period, significant disturbances especially those caused by humans might have degraded the community.

### The relationship between soil pollen profile and current vegetation

Similar to a previous study by Pan *et al*.^23^, our results also reveal that abundant pollen survive in the surface soils of forests in subtropical China. Although the dominant in the surface soil generally reflect the current plant community (Table 1), in both near-natural and artificial forests at our study sites, the similarity between the surface soil pollen and current vegetation was relatively low (<30%). Previous studies also reported low similarity between the soil seed bank and existing vegetation (ca. 40%) in this area^23^,^24^,^25^. Generally, sites with a long history contain relatively less information about present vegetation^26^. The information in soil pollen is much older than that in soil seed banks, leading to lower similarities between soil pollen and current vegetation (Table 5). The taxa that appear in both pollen diagram and seed banks should receive more attention in restoration practice under climate change, because these taxa are relatively stable during long periods of climatic and environmental changes.

In addition, the R values of individual pollen taxa varied across study sites and vegetation types mainly because of the differences in pollen deposition^27^,^28^,^29^. The R values might also be influenced by individual size which had a close relationship with pollen production. At our study sites, many pollen taxa were detected in the surface soil even though the taxa were not present in existing vegetation, including *Pinus* in bare land, *Eurya* in broadleaved forest, *Hicriopteris* in pine and broadleaved mixed forest and coniferous forest, *Palhinhaea cernua* in eucalyptus plantation, and *Artemisia* in broadleaved plantation. The dominance of *Pinus* pollen in bare land might indicate an extra-local origin for pine pollen, especially considering that *Pinus* has a high R value and it is therefore over represented in the pollen rain, and also that pine pollen can be dispersed over long distances. Other pollen types may come from adjacent areas or the relevant individuals aboveground had been replaced by other species during succession^30^,^31^,^32^. On the other hand, pollen of some species in the current vegetation were not detected in the surface soil; and such species included *Pterospermum Schreber* and *Blastus* in broadleaved forest, *Psychotria* in pine and broadleaved mixed forest, and *Gardenia Ellis* in broadleaved plantation. This might be due to (1) a low pollen production by these species; (2) the thick layer of litter in near-natural forest, (3) unsuitable preservation environment related to degradation of the pollen and spores in the soil, and/or (4) the small size of the sampled area relative to the area of vegetation.

Overall, these results indicate that (1) there is a significant difference between soil pollen and aboveground vegetation; (2) the dominant pollen can provide useful information for selecting reference ecosystems; and (3) there are also some uncertainty in the relationship between pollen and vegetation, indicating that the soil pollen-vegetation relationship should be carefully evaluated and applied.

### Implications for forest restoration of the degraded lands

The pollen spectrum in broadleaved forest indicates a shift from pine forest to broadleaved forest, which is consistent with near-natural succession of subtropical forests in South China. Pollen from *Pinus* was dominant in the 40–80 cm layer, and the percentage of *Castanopsis* was small but Poaceae pollen was abundant. The pollen spectrum in the 40–80 cm layer in bare land was very similar to that in the broadleaved forest, indicating that they might have had a similar regional development history. The similar pollen spectra in bare land and broadleaved forest indicate that the bare land is experiencing natural succession and does not represent a novel ecosystem.

The Society for Ecological Restoration International Science & Policy Working Group (2004)^6^ indicated that fossil pollen, charcoal, tree ring data, and rodent middens could be used to describe reference ecosystems. In certain cases, information from multiple sources can be integrated for selecting or establishing a reference ecosystem. For bare land in areas that lack other information, however, fossil pollen in soil profile may be especially valuable^33^. In subtropical China, selecting a proper reference ecosystem based on known development history is critical for restoring the degraded or bare lands. For example, pollen research from Jiang *et al*.^34^ indicated that herbs were dominant both in the cold–dry LGM and the warm–humid early–mid Holocene on the Chinese Loess Plateau. This explains why directly planting trees on the Chinese Loess Plateau often has had negative ecological outcomes, such as low tree survival, increased soil erosion, exacerbated water shortages, and deep-soil desiccation. Following Jiang *et al*.^34^, ecological restoration on the Chinese Loess Plateau should emphasize herbs rather than trees and shrubs.

In our study, pollen of *Gironniera* and Rutaceae, two commonly observed taxa in the climax community monsoon evergreen broad leaved forests, were detected in the soil profile (40–80 cm layer) of bare land. This layer also contained pollen from *Rhodomyrtus*, Myrsinaceae, *Lonicera, Cyclobalanopsis*, and *Schima*. These observations indicate that a monsoon evergreen broadleaved forest existed in this area between ca. 1000–2000 years ago and that it is possible to restore the bare lands to monsoon evergreen broadleaved forest. In addition, bare land contained dominant pollen from Pinus and Poaceae, and less dominant pollen of *Lonicera Linn*., *Casuarina Adans, TremaLour*, and *Quercus*. This observation suggests that early restoration of such land in south China may benefit from using such taxa.

In the soil pollen profile (0–80 cm) of broadleaved forest, some “stable taxa” defined as the species with pollen existed in all soil layers (i.e., in soils of all ages) deserve special attention. Such taxa included species from *Pinus, Castanopsis, Gironniera*, Rutaceae, *Helicia, Randia*, Poaceae, *Dicranopteris*, and *Pteris* (Fig. 3 and Table 6) and had existed for at least 2552 years BP in the area (Fig. 3 and Table 2). During that period, the temperature fluctuation in the Northern Hemisphere reached 1 °C^35^,^36^. These “stable taxa” probably have contributed to the stability of the forest succession in South China. Pollen of *Baeckea, Chenopodiaceae, Artemisia*, and *Brassicaceae*, whose distributions are usually limited to the temperate zone, appeared in the top soil layers in broadleaved forest but the individual plants of these taxa were not observed in present vegetation. This indicates that the climate in subtropical China previously suitable for temperate plants may have shifted northward during last ~100 years, at least in part due to recent global warming^37^.

Historical data are clearly useful for identifying key ecological legacies and for selecting reference ecosystems under rapid environmental changes^38^. Returning the degraded lands to the natural successional trajectory based on the historical data represents an improved approach for restoration in subtropical China^18^,^39^.

## Materials and Methods

### Study sites

The study sites were located at the Dinghushan Natural Reserve (23°09′–23°11′N, 112°30′–112°33E) and the Heshan National Field Research Station of Forest Ecosystem (22°34′N, 112°50′E) in Guangdong, South China. Although climate and soil conditions are similar at these two sites, Dinghushan has more near-natural vegetation whereas most forests at Heshan are plantations on degraded land. There are also some bare lands in Heshan. The climate in this region is subtropical monsoon, and the predominant soil types are lateritic red-earth and yellow-earth^40^. The mean annual rainfall is 1534–1927 mm, and nearly 80% of the precipitation occurs in the wet season from April to September. The mean annual temperature ranges from 20.9 to 22.5 °C, and the mean relative humidity is 80.8%^19^. The regional climax plant community is a subtropical monsoon evergreen broadleaved forest; the four major successional stages are grassland, pine forest, mixed-pine forest, and broadleaved forest^22^.

We selected a total of eight communities for this study on soil pollen and aboveground vegetation. Three near-natural communities were at Dinghushan: a coniferous forest (CF), a pine-broadleaved mixed forest (MF), and a monsoon evergreen broadleaved forest (BF) which represent three natural successional stages in subtropical China. Four artificial communities (plantations) were at Heshan: a mixed broadleaved plantation (BP), a mixed-coniferous plantation (CP), a eucalyptus plantation (EP), and a mixed-legume plantation (LP). One selected degraded bare land was also at the Heshan site. The selected near-natural and artificial communities are the most common community types in South China. The dominant tree species and properties of the communities are described in Table 7.

### Vegetation sampling

Twenty 20 m × 20 m plots were established in broadleaved forest and pine-broadleaved mixed forest, and nine plots (10 m × 10 m) were established in each plantation (Legume plantation, coniferous plantation, broadleaved plantation, and Eucalyptus plantation) for tree surveys. In each of these plots, one 5 m × 5 m subplot and one 1 m × 1 m subplot were randomly selected to survey shrubs and herbs, respectively. In the coniferous forest, ten 20 m × 20 m plots were established for tree surveys due to its smaller area, and in each of these plots, six 5 m × 5 m subplots and six 1 m × 1 m subplots were established to survey shrubs and herbs, respectively. All tree species (DBH ≥ 1 cm) in every 20 m × 20 m and 10 m × 10 m plot, all shrubs in every 5 m × 5 m subplot, and all herbs in every 1 m × 1 m subplot were identified and recorded. The total sampled areas of near-natural forest and plantations were 20,000 m^2^ and 4000 m^2^, respectively. The investigated areas for each vegetation type met the minimal area required for vegetation survey in the subtropics^41^. The detailed vegetation data were provided in previous studies in 2013 and 2014^18^,^19^.

### Soil pollen and ^14^C dating

Six 10 m × 10 m plots were established on a transect that was parallel to the slope at each near-natural forest and plantation. Five surface soil cores (5 cm deep and 4 cm in diameter) were collected from the center and four corners of each plot. After the litter was removed, the samples were mixed. Two soil profiles to 80 cm depth were excavated in broadleaved forest (regional climax) and bare land. In each profile, soil samples were collected at depths of 0–5, 5–10, 10–20, 20–40, and 40–80 cm, respectively. The depth intervals were not at a constant resolution, because there were little pollen existed below the 40-cm depth in this area. After the samples were air-dried and visible roots and stones were removed, the samples were passed through a 1-mm sieve to remove rootlets and coarse sands.

#### Soil pollen

The soil treatment and pollen identification were conducted in Nanjing Institute of Geology and Palaeontology, Chinese Academy of Sciences; China. Soil samples were treated with HF to extract pollen following Fægri *et al*.^11^. Subsamples (2 g) of air-dried soil were weighed, and one *Lycopodium* spore tablet (27673 ± 200 spores) was added as a tracer to each sample, which was then treated with 10% HCl, 10% NaOH, and 40% HF before it was passed through a 7-μm mesh screen. The pollen grains, which were collected on the screen, were identified and counted with the aid of an optical microscope at 400× magnification. More than 200 grains from each sample were counted.

#### ^*14*^C dating

The ^14^C dating analysis of bare land was conducted in Guangzhou Institute of Geochemistry, the Chinese Academy of Sciences. The ages of the different layers of the soil profile in the broadleaved forest and bare land were determined by ^14^C dating. Soil samples were first dried, and the cohesive carbonate was eliminated with 2 M HCl. After the soil organic carbon was transformed into CO_2_ in a 850 °C muffle furnace, the soil CO_2_ was purified using liquid N_2_ and liquid N_2_ ethanol traps in the vacuum system and then measured using an AGI LENT-6890N gas chromatograph^42^. The ^*14*^*C* dating of the broadleaved forest followed Shen *et al*.^43^.

### Coefficient of similarity

The representativeness of pollen was calculated as follows^27^:


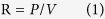


where R is the representativeness of the pollen; *P* is the percentage of pollen in soil samples, and *V* is the percentage of individuals in the community. R > 1 indicates over representation; R < 1 indicates under representation; and R = 1 indicates moderate representation.

The similarity between the surface soil pollen and the current plant community was calculated using the Sorensen Index and Jaccard Index:


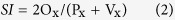



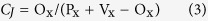


where *SI* is the Sorensen index and *C*_*J*_ is the Jaccard Index, both representing the similarity between the soil pollen and vegetation; *O*_*X*_ is the number of overlapping taxa between soil pollen and vegetation; *P*_*X*_ is the number of taxa observed in the soil pollen; and *V*_*X*_ is the number of taxa in current vegetation. A *t*-test was used to analyze the differences in *SI* and *C*_*J*_ values between near-natural forest and plantation.

## Additional Information

**How to cite this article**: Sun, Z. *et al*. To What Extent Local Forest Soil Pollen Can Assist Restoration in Subtropical China? *Sci. Rep.* **6**, 37188; doi: 10.1038/srep37188 (2016).

**Publisher’s note**: Springer Nature remains neutral with regard to jurisdictional claims in published maps and institutional affiliations.

## Figures and Tables

**Table 1:** The characteristics of pollen in the surface soils in forest communities in subtropical China.

Site	Common pollen (%)	Dominant pollen (%)		
		Tree	Herb	Fern
D-C	—	*Pinus* (63.8)	Poaceae (30.3)	*Dicranopteris* (360)
D-Z	*Rhodomyrtus* (5.8) *Myrsinaceae* (5.2)	*Pinus* (63.4)	Poaceae (14.2)	*Dicranopteris* (42.9)
D-H	*Pinus* (28.4) *Myrsinaceae* (4.3)	*Castanopsis* (49.2)	Poaceae (10.2)	*Dicranopteris* (53.1)
D-J	*Gironniera subaequalis* (5.1) *Engelhardia* (4.1)	*Castanopsis* (62.4)	Poaceae (0.6)	*Dicranopteris* (5.7)
H-Z	*Trema* (8.8) *Ilex* (8.0)	*Pinus* (24.4)	Poaceae (34.9)	*Dicranopteris* (68.7)
H-X	*Castanopsis* (8.2) *Myrsinaceae* (8.9)	*Pinus* (22.6)	Poaceae (29.6)	*Dicranopteris* (318)
H-A	*Ilex* (12.6) *Trema* (4.5)	*Myrsinaceae* (39.3)	Poaceae (8.9)	*Dicranopteris* (43.8)
H-D	*Myrsinaceae* (16.0) *Mallotus* (11.2)	*Ilex* (29.2)	Poaceae (14.7)	*Dicranopteris* (40.2)

Note: CF = coniferous forest; MF = pine and broadleaved mixed forest; BF = broadleaved forest; CP  = coniferous plantation; BP = broadleaved plantation; EP = eucalyptus plantation; LP = legume plantation. Percentages are relative to the total tree and herb pollen in each sample, and the percentage showed in the table is the average value.

**Table 2:** The ^14^C dating (a BP) of soils from the broadleaved forest and bare land in Guangdong, South China.

Depth (cm)	^14^C dating age (years BP)	
	Site BF*	BL
0–5	—	—
5–10	—	—
10–15	761 ± 68	1040 ± 15
15–20	485 ± 66	590 ± 23
20–30	791 ± 67	505 ± 20
30–40	1315 ± 75	360 ± 18
40–50	1959 ± 75	1405 ± 20
50–60	2064 ± 73	1660 ± 16
60–70	2557 ± 73	2375 ± 25
70–80	2047 ± 73	1130 ± 20

Note: BF = broadleaved forest; BL = bare land.

*Data cited from Shen *et al*.^43^.

**Table 3:** Pollen representativeness (R value) of main taxa in near-natural and artificial communities at the two study sites.

Pollen types	R value in the indicated community							
	BF	MF	CF	CP	EP	BP	LP	Average
*Ficus*	—	—	0.02	—	—	—	—	0.02
Rutaceae	0.06	0.21	—	—	—	—	—	0.14
*Mussaenda*	—	—	—	—	—	—	0.26	0.26
*Adiantum*	—	—	—	0.37	—	—	—	0.37
*Glochidion*	—	—	0.57	—	—	—	—	0.57
*Lindsaeaceae*	—	—	—	—	—	0.10	1.29	0.69
*Ilex*	0.60	—	—	0.70	1.48	0.03	0.99	0.76
*Phyllantus*	—	—	—	—	—	—	0.77	0.77
*Melastoma*	1.37	1.52	2.55	1.32	—	0.25	2.23	1.54
*Schima*	0.84	0.05	5.74	—	—	0.18	—	1.70
*Rhodomyrtus*	—	—	—	—	—	3.56	0.36	1.96
*Desmos chinensis*	2.39	—	—	—	—	—	—	2.39
Myrsinaceae	0.05	0.21	—	—	—	—	8.00	2.75
*Elaeocarpus*	2.87	—	—	—	—	—	—	2.87
*Eucalyptus*	—	—	—	—	3.56	—	—	3.56
*Pteris*	—	4.05	—	—	—	—	—	4.05
*Gironniera subaequalis*	4.33	—	—	—	—	—	—	4.33
*Pinus*	—	6.65	0.92	6.81	—	—	—	4.79
*Mallotus*	0.07	6.08	0.42	1.22	—	6.71	15.21	4.95
*Baeckea frutescens*	—	8.11	4.30	—	—	—	—	6.21
*Dicranopteris*	—	14.46	8.93	19.63	1.32	9.67	1.56	9.26
*Castanopsis*/*Lithocarpus*	19.85	3.14	—	—	—	—	—	11.49
*Trema*	19.13	—	12.91	—	—	5.45	7.22	11.18
*Poaceae*	1.07	4.51	36.46	0.94	6.73	2.93	28.10	11.53
*Acacia*	—	—	—	—	1.53	—	33.67	17.60
*Engelhardia*	18.65	—	—	—	—	—	—	18.65

Note: BF = broadleaved forest; MF = pine and broadleaved mixed forest; CF = coniferous forest; CP = coniferous plantation; EP = eucalyptus plantation; BP = broadleaved plantation; LP = legume plantation.

**Table 4:** The similarities between pollen in the surface soil (0–5 cm depth) and current vegetation in South China.

Status	Community	Ox	Px	Vx	Sorensen Index (*SI*)	Jaccard Index (*C*_*J*_)
Near-natural	BF	12	34	100	0.179	0.098
	MF	10	28	63	0.220	0.123
	CF	9	37	36	0.247	0.141
	Average	10.3	33.0	66.3	0.215	0.121
Plantation	CP	6	35	32	0.179	0.098
	EP	4	31	27	0.138	0.074
	BP	8	46	40	0.186	0.103
	LP	11	39	47	0.256	0.147
	Average	7.3	37.8	36.5	0.190	0.105
Mean		8.6	35.7	49.3	0.201	0.112

Note: BF = broadleaved forest; MF = pine and broadleaved mixed forest; CF = coniferous forest; CP = coniferous plantation; EP = eucalyptus plantation; BP  = broadleaved plantation; LP = legume plantation. *O*_*X*_ is the number of overlapping taxa between the soil pollen and vegetation; *P*_*X*_ is the number of taxa observed in the soil pollen; and *V*_*X*_ is the number of taxa in current vegetation.

**Table 5:** Similarities as indicated by the Sorensen Index between pollen in the surface soil (0–5 cm) and current vegetation and between the soil seed bank and current vegetation in the study sites.

Site and forest type	Sorensen Index (*SI*)	
	Pollen & Vegetation	Seed bank & Vegetation
Dinghushan-Near-Natural forests	0.22	0.44*
Heshan-Plantations	0.19	0.34**
Average	0.21	0.39

*(Shi *et al*.)^25^.

**(Wang *et al*.^24^; Wang *et al*.^44^).

**Table 6:** The pollen counts of “stable taxa” in broadleaved forest soil profiles in subtropical China.

Depth (cm)	Pollen counts of different taxa								
	*Pinus*	*Castanopsis*/*Lithocarpus*	*Gironniera*	Rutaceae	Poaceae	*Helicia*	*Monolete*	*Dicranopteris*	*Pteris*
0–5	12	181	44	56	11	1	1	25	1
5–10	10	409	21	76	1	3	11	38	2
10–20	8	279	10	52	2	3	12	48	1
20–40	4	218	32	28	1	1	19	102	1
40–80	28	8	45	38	3	1	152	390	94

**Table 7:** The vegetation characteristics at the two study sites in subtropical China.

Site	Community	Year after protection (years)	Dominant species in tree/shrub layer	Species number in tree layer
Dinghushan	Coniferous forest (CF)	29	Tree layer: *Pinus massoniana*	33
Dinghushan	Pine and broadleaved mixed forest (MF)	~60	Tree layer: *Pinus massoniana, Castanopsis chinensis, Schima superba*	55
Dinghushan	Monsoon evergreen broadleaved forest (BF)	~400	Tree layer: *Cryptocarya concinna, Cryptocarya chinensis*	88
Heshan	Legume tree species mixed plantation (LP)	28	Tree layer*: Acacia auriculiformis*., *Acacia mangium*., *Erythrophleum fordii*.	11
Heshan	Coniferous tree species mixed plantation (CP)	28	Tree layer: *Pinus massoniana*., *Cunninghamia lanceolata*.	13
Heshan	Native tree species mixed plantation (BP)	28	Tree layer: *Schima wallichii*., *Schima superba, Castanopsis hystrix*., *Michelia macclurei*.	21
	Eucalyptus plantation (EP)	28	Tree layer: *Eucalyptus urophylla, Eucalyptus exserta*.	19
Heshan	Bare land (BL)	>20	—	—
